# Genetic Variants in the STMN1 Transcriptional Regulatory Region Affect Promoter Activity and Fear Behavior in English Springer Spaniels

**DOI:** 10.1371/journal.pone.0158756

**Published:** 2016-07-08

**Authors:** Xiaolin Ding, Jin Hu, Hanying Zhang, Yinxue Xu

**Affiliations:** 1 Department of Animal Genetics, Breeding and Reproduction, College of Animal Science and Technology, Nanjing Agricultural University, Nanjing, Jiangsu Province, People’s Republic of China; 2 Pharmacology Department, R&D center, Nanjing Sanhome Pharmaceutical Co. LTD, Nanjing, Jiangsu Province, People’s Republic of China; National University of Ireland Galway, IRELAND

## Abstract

Stathmin 1 (*STMN1*) is a neuronal growth-associated protein that is involved in microtubule dynamics and plays an important role in synaptic outgrowth and plasticity. Given that STMN1 affects fear behavior, we hypothesized that genetic variations in the *STMN1* transcriptional regulatory region affect gene transcription activity and control fear behavior. In this study, two single nucleotide polymorphisms (SNPs), g. -327 A>G and g. -125 C>T, were identified in 317 English Springer Spaniels. A bioinformatics analysis revealed that both were loci located in the canine *STMN1* putative promoter region and affected transcription factor binding. A statistical analysis revealed that the TT genotype at g.-125 C>T produced a significantly greater fear level than that of the CC genotype (*P* < 0.05). Furthermore, the H4H4 (GTGT) haplotype combination was significantly associated with canine fear behavior (*P* < 0.01). Using serially truncated constructs of the *STMN1* promoters and the luciferase reporter, we found that a 395 bp (−312 nt to +83 nt) fragment constituted the core promoter region. The luciferase assay also revealed that the H4 (GT) haplotype promoter had higher activity than that of other haplotypes. Overall, our results suggest that the two SNPs in the canine *STMN1* promoter region could affect canine fear behavior by altering *STMN1* transcriptional activity.

## Introduction

Police dogs play a unique role in preventing and cracking down on crime [1]. In the Nanjing Police Dog Institute of the Ministry of Public Security of the People’s Republic of China, the training records of English Springer Spaniels show that many puppies are unable to be trained into working dogs because they are fearful. Fear can be affected by genetic, environmental [2, 3] (or their interaction) and epigenetic factors [4]. As an emotional state, fear can protect the dog by defensive behavior or escape once it perceived danger [5, 6]. Fear can be triggered by various stimuli, such as novel or intense stimuli, special evolutionary danger, social interactions with conspecifics, and conditioned stimuli [7]. Fear-based behavioral responses include aggressive behavior [8, 9], avoidance responses [10], flight [11], withdrawal [12], and immobility (freezing or crouching) [13, 14]. As fearful subjective states cannot be directly measured by self-report in dogs, the experience of fear is based on proxy measures of behavioral responses. Fearful behavior in dogs can be assessed by objective behavioral testing or subjective questionnaire surveys. In general, few differences are found between behavioral tests and questionnaire surveys in the consistency of assessing the fearfulness of a dog [15–17]. The genetic background of fear is polygenic and most likely involves genes associated with different cellular processes and pathways, but the specifics remain unclear [18]. Thus, further studies to identify specific genes involved in the formation and expression of fear are needed.

The microtubule destabilizers Stathmin family, includes STMN1, STMN2 (SCG10: superior cervical ganglion-10 protein), STMN3 (SCG10-like protein) and STMN4 (stathmin-like protein B3) [19–22]. They have a common tubulin binding site which can destabilize MT and sequester tubulin heterodimer. *STMN1* is widely distributed and localized in the cytosol [23], whereas *STMN2*, *STMN3* and *STMN4* are neuron-specific and located on membranes of developing (*STMN2* and *STMN3*) and mature (*STMN4*) nerve cells [19–22]. STMN1 expresses highly in thalamus, cortex and lateral amygdala and regulates MT dynamics, synaptic growth and plasticity [24–26].

Several studies have reported that *STMN1* is closely related to fear level in humans and animals. Two single nucleotide polymorphisms (SNPs) (rs182455 and rs213641) in the transcriptional control region of STMN1, were found to affect fear and anxiety responses in humans [27]. The results of a subsequent study showed that the rs182455 C-allele alters the cognitive-affective processing of healthy people and could affect fear processing [28]. In mice, knocking out *STMN1* led to abnormal spike-timing-dependent long-term potentiation and defective memory on fear conditioning [29–31].

However, genetic variations in the *STMN1* transcriptional regulatory region have not yet been reported in dogs. Moreover, the distribution, frequency, and function of the *STMN1* variations are unclear, and the regulation of *STMN1* transcriptional activity by the promoter region remains to be revealed. Therefore, the present study investigated potentially functional genetic variations in the *STMN1*promoter and explored whether these genetic variations could affect gene transcriptional activity and, consequently, fear behavior.

## Materials and Methods

### Sample collection

A total of 317 English Springer Spaniels from the Nanjing Police Dog Institute of the Ministry of Public Security were employed in this study (Nanjing, Jiangsu Province, China). The ages of these dogs ranged from 6 to 10 months with 142 males and 175 females. Blood samples (2 mL) were collected from forelimb vein by a veterinarian and stored at −20°C for DNA extraction. Heart, liver, spleen, lung, kidney, pancreas, muscle, medial prefrontal cortex (mPFC), midbrain, cerebellum, brainstem, amygdala, hippocampus, hypothalamus, hypophysis, spinal cord, medulla oblongata, and olfactory bulb tissues were collected from 15 dogs, within 30min of euthanasia by overdose of pentobarbital, frozen and stored in liquid nitrogen until total RNA extraction. All experimental procedures and sample collections were performed according to the Regulations for the Administration of Affairs Concerning Experimental Animals (Ministry of Science and Technology, China; revised in June 2004) and approved by the Ethics Committee of Nanjing Agricultural University. All efforts were made to minimize the number of animals used and their suffering.

### Fear behavioral test

The fear level test consisted of five separate subtests: the affability and handling test [17], floor test, gunshot test [32], umbrella test, and aluminum food bowl test [17, 33]. The tests were administered to 317 dogs, and were conducted outdoors in a specific order. All dogs were scored from 1 to 5 according to their fear reaction across all subtests, where 1 = no fear, 2 = slight fear, 3 = obvious fear, 4 = very frightened, and 5 = terrified. The results of behavioral tests are presented in S1 Table.

### RNA preparation and STMN1 gene expression in different tissues

Total RNA was extracted from heart, liver, spleen, lung, kidney, pancreas, muscle, mPFC, midbrain, cerebellum, brainstem, amygdala, hippocampus, hypothalamus, hypophysis, spinal cord, medulla oblongata, and olfactory bulb tissues using TRIzol reagent (Invitrogen, Carlsbad, CA, USA) as previous described [34]. RNA purity was determined by the ratio of OD value at 260/280 nm (1.8–2.0). Total RNA was reversely transcribed in a 20 μL reaction mixture at 25°C for 10 min, 42°C for 30 min, and 85°C for 5 min with 5× QRT SuperMix (Vazyme, Nanjing, China). The primer pair sequences for the target genes (Table 1) were generated by Primer Premier 5 software. Real time quantitative reverse transcription polymerase chain reaction (qRT-PCR) was performed in a 20 μL system including 2 μL cDNA (50–100 ng/μL), 10 μL AceQ^™^ qPCR SYBR Green Master Mix (TransGen, Beijing, China), 0.4 μL ROX Reference Dye II, 0.8 μL (10 μM) of each primer, and 6 μL RNase-free water. PCR program is the same as previously described [35]. The qRT-PCR data were analyzed using the 2^−ΔΔCt^ method for mRNA quantification.

**Table 1:** Primers used in this study.

Primer	Length (bp)	Primer sequence (5′-3′)	Tm (°C)
P-F1	2,387	GG*GGTACC*ATGGTTGGGGAGGGTTGGT	61
P-F2	1,696	GG*GGTACC*CGGTGGCACAGCAGTTTAG	58
P-F3	1,293	GG*GGTACC*TGACAAGACCAAGGCACGAG	61
P-F4	980	GG*GGTACC*GACAACAGCCTAGCTCGTTTG	58
P-F5	675	GG*GGTACC*GCTCTGCCCCTATCCAAAG	58
P-F6	395	GG*GGTACC*TGTGGCAGGACTAGGCATCT	58
P-R	-----	CCG*CTCGAG*ACACAAATGAGCACGGAACC	------
F1/R1	427	TCTCATGGGCAAAGATAG/GCTTGTGGGTTTGTTGTT	56
F2/R2	405	GGACTAGGCATCTAACAAC/GGACTAGGCATCTAACAAC	56
F3/R3	207	ATCCGTCCCAGAATTCC/TCTTCCGCCATCTTACTG	60
β-actin-F/R	121	GGGCCAGAAGGACTCCTACG/GTGCCAGATCTTCTCCATGTC	60

### DNA preparation and screening for genetic variants in the promoter

Genomic DNA was extracted from blood samples using Proteinase K digestion, phenol chloroform extraction, and ethanol precipitation. The 317 total dogs were classified into two groups (n = 12) with high- or low-fear levels respectively. Two DNA pools for the high- and low-fear groups were prepared by mixing equal amounts of genomic DNA from 12 dogs. The transcription start site was marked as +1. The 5′-flanking transcriptional regulatory region (−2,034 nt to +83 nt) upstream in *STMN1* was sequenced and scanned with the P-F1/R primer (Table 1). The SNPs were identified by DNA sequence alignment between the high and low-fear groups using DNASTAR software (ver. 5.0; DNASTAR Inc., Madison, WI, USA).

### Genotyping of the polymorphisms

The sequencing results disclosed two genetic variations (g. -327 A>G and g. -125 C>T) in the canine *STMN1* promoter. The g. -327 A>G and g. -125 C>T genotypes were examined using the polymerase chain reaction single-strand conformation polymorphism (PCR-SSCP) technique. The F1/R1 and F2/R2 primers (Table 1) were designed to amplify the 427 bp and 405 bp PCR products harboring g. -327 A>G and g. -125 C>T, respectively. PCR amplifications system contains 100 ng DNA template, 10 μL 2× Taq Premix, 0.5 μL of each primer (10 μM), and double distilled H_2_O up to 20 μL. The cycling protocol is the same as previously described [36]. Aliquots of 10 μL of the PCR products were mixed with 10 μL denatured solution, incubated at 98°C for 10 min, and chilled on ice. The denatured DNA was subjected to 8% polyacrylamide gel electrophoresis in 1× TBE buffer and separated at a constant 160 v for 16 h. The gel was stained with 0.1% silver nitrate and visualized with 2% NaOH solution (containing 0.1% formaldehyde) [37]. The PCR products from the different PCR-SSCP genotypes were sequenced.

### Cloning and construction of the *STMN1* reporter plasmids

To evaluate the promoter activity of the *STMN1* transcriptional regulatory region, we conducted serial truncations of the *STMN1* promoter fragment, ranging from −2,304 nt to +83 nt, to analyze reporter construct activity. The six *STMN1* primer pairs are listed in Table 1. The forward and reverse primers contained the *Kpn* I and *Xho* I restriction sites, respectively. The PCR products were purified, and the target DNA fragments were digested with the *Kpn* I and *Xho* I restriction enzymes and cloned into multiple cloning sites (*Kpn* I and *Xho* I) of the pGL3-basic vector (Promega, Madison, WI, USA). The recombinant plasmids were confirmed by sequencing and denoted pGL3-F1, -F2, -F3, F4, F5, and F6, respectively. To compare the transcriptional activities of the *STMN1* promoters between various haplotypes, the regulatory regions from −592 nt to +83 nt containing the H1(AC), H2(AT), H3(GC), and H4(GT) haplotypes were amplified with the P-F5/R primer pair (Table 1). The amplified products were digested with the *Kpn* I and *Xho* I restriction enzymes and subsequently cloned into multiple cloning sites of the pGL3-basic vector. The constructs were denoted pGL3- H1, -H2, -H3, and -H4, respectively.

### Luciferase assay for promoter activity

Before transfection, 293T cells were seeded in 12-well plates. When cells had grown to 70–80% confluence, 0.05 μg pRL-TK vector (Promega) and 1 μg pGL3 vector were co-transfected with 3 μL Lipofectamine^™^ 2000 reagent (Invitrogen) following the manufacturer’s instructions. The pRL-TK vector is an internal control to correct for differences in transfection and harvesting efficiency. The luciferase assay was conducted as the manufacturer instructed (Promega). Firefly and Renilla luciferase activities were measured with a luminometer (Modulus; Turner Biosystems, Sunnyvale, CA, USA). Promoter activity is reported in relative light units and normalized against thatof the empty pGL3-basic vector. All transfections were performed in triplicate and repeated at least three times in independent experiments.

### Statistical analysis

Genotype frequencies, allelic frequencies, effective number of alleles, heterozygosity, polymorphism information content (PIC), and the chi-square test of goodness-of-fit for the Hardy-Weinberg equilibrium law were calculated using PowerMarker V3.25 [38, 39]. Statistical comparisons were made by one-way ANOVA followed by Duncan’s multiple comparisons test using SPSS for Windows software (ver. 18.0; SPSS Inc. Chicago, IL, USA). *P*-values < 0.05 were considered significant. All data are expressed as means ± standard error.

## Results

### *STMN1* expression analysis and correlation with fear behavior in English Springer Spaniels

**Fig 1:**
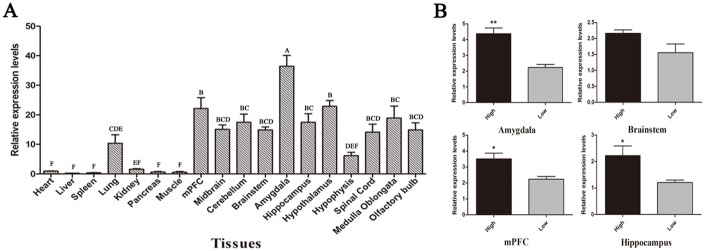
*STMN1* gene expression in different canine tissues. (A) Canine Stathmin 1 (*STMN1*) mRNA expression profiles in 18 tissue types, determined in triplicate. The mean heart level was set to 1. Different capital letters on the bars indicate significant differences (*P <* 0.01) (B) *STMN1* expression level in the low and high fear level groups (n = 5/group) in the amygdala, brainstem, medial prefrontal cortex (mPFC), and hippocampus, respectively. The mRNA levels were normalized to β-actin mRNA levels. *P < 0.05; **P < 0.01.

Real-time PCR was performed to examine *STMN1* expression in 18 tissues (heart, liver, spleen, lung, kidney, pancreas, muscle, mPFC, midbrain, cerebellum, brainstem, amygdala, hippocampus, hypothalamus, hypophysis, spinal cord, medulla oblongata, and olfactory bulb) of the dogs. High levels of *STMN1* transcripts were detected mainly in brain tissues (amygdala, brainstem, mPFC, and hippocampus), and low levels were detected in heart, liver, spleen, kidney, pancreas, and muscle (Fig 1A). Four brain tissues were selected to compare *STMN1* transcription levels between the high- and low-fear level dog groups. The RT-qPCR assay revealed that the high-fear level group expressed higher STMN1 mRNA levels than those of the low-fear level group (*P* < 0.05) in the amygdala, mPFC, and hippocampus (Fig 1B), but not in the brainstem. A significant correlation was detected between *STMN1* mRNA levels in the amygdala, mPFC, and hippocampus and fear behavior of the dogs.

### Two SNPs were identified in the *STMN1* transcriptional regulatory region

**Fig 2:**
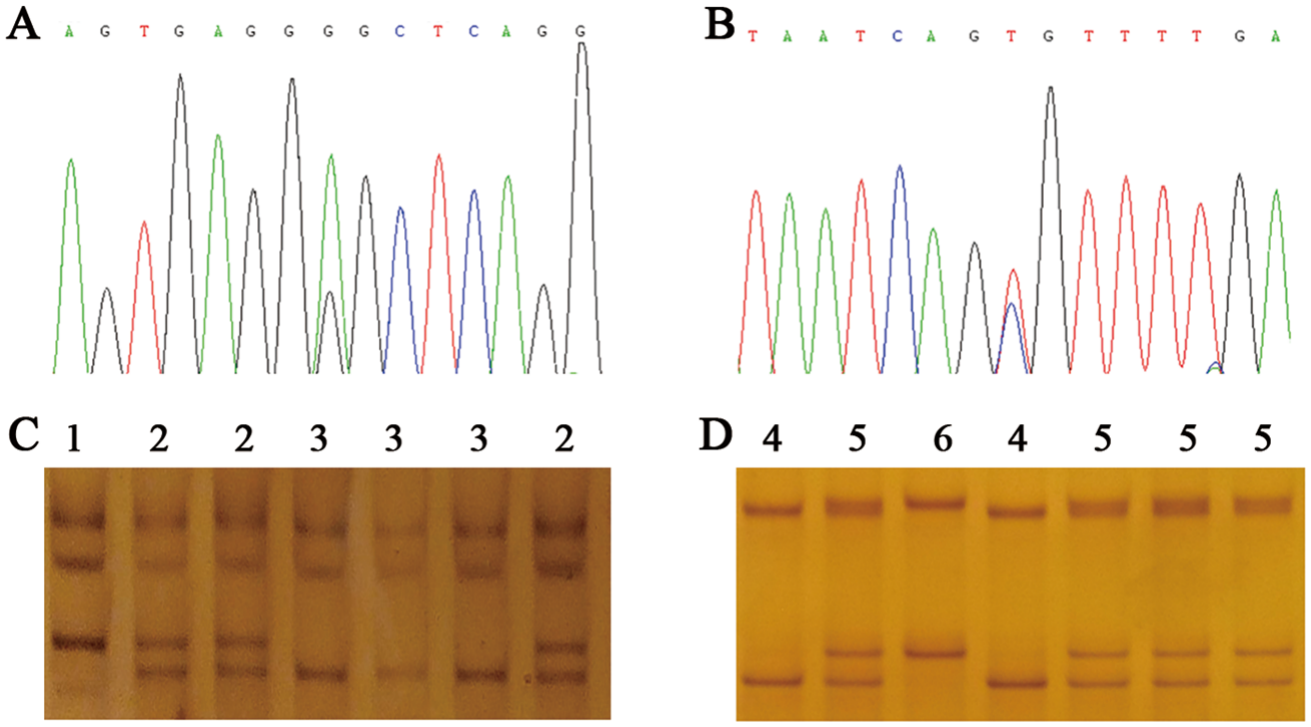
Genetic variation in the canine *STMN1* 5′-flanking region sequence and polymerase chain reaction single-strand conformation polymorphism (PCR-SSCP) patterns. (A) Sequencing diagrams using DNA pools of g. -327 A>G. (B) Sequencing diagrams using DNA pools of g. -125 C>T. (C) Electrophoresis patterns of PCR-SSCP at position g. -327 A>G, 1 = GG, 2 = AG, and 3 = AA. (D) Electrophoresis patterns of PCR-SSCP at position g. -125 C>T, 4 = CC, 5 = CT, and 6 = TT.

We assumed that genetic variations in the *STMN1* transcriptional regulatory region might affect its expression level. Hence, we sequenced a 2,387 bp segment from the *STMN1* transcriptional regulatory region in English Springer Spaniels and identified two genetic variations (g. -327 A>G and g. -125 C>T) (Fig 2A and 2B). The two SNPs were further genotyped in a population of 317 dogs using the PCR-SSCP technique (Fig 2C, 2D and S1 Table). The genotyping results verified the DNA sequencing results. We found that the A and C alleles were the predominant g. -327 A>G and g. -125 C>T alleles, respectively. The chi-square test showed that the two SNPs were in Hardy—Weinberg equilibrium (*P* > 0.05) within the analyzed population. According to the PIC classification (PIC value < 0.25, low polymorphism; 0.25 < PIC value < 0.5, intermediate polymorphism; and PIC value > 0.5, high polymorphism), all loci exhibited intermediate polymorphism at the *STMN1* in the analyzed population (Table 2).

**Table 2:** Genotype distribution, allelic frequencies, and genetic diversity of the two single nucleotide polymorphisms (SNPs) in canine stathmin 1 (*STMN1*).

SNP	Sample size	Genotype frequencies	Allele frequencies	He	Ne	PIC	p-value (χ^2^, HWE)
g. -327 A>G	317	AA (0.3028) AG (0.4732) GG (0.223)	A (0.5394) G (0.4606)	0.4732	1.9019	0.3734	0.3696
g. -125 C>T	317	GG (0.2776) CT (0.5426) TT (0.1798)	C (0.5488) T (0.4511)	0.5426	2.1862	0.3726	0.0858

HWE, Hardy-Weinberg equilibrium.

### Association between the two *STMN1* SNPs and fear behavior in English Springer Spaniels

The effects of the two SNPs on the fear level of 317 dogs are summarized in Table 3. The results suggest that the g. -125 C>T polymorphism was associated with fear level (*P* < 0.05). Animals with the g. -125 C>T TT genotype had an obviousgreater fear level than those with the CC genotype (*P* < 0.05), whereas the g. -327 A>G genotype had no relationship with fear (*P* > 0.05).

**Table 3:** Least squares mean (LSM) ± standard error for the fear level of different *STMN1* SNPs in English Springer Spaniel.

SNP	Genotype	Sample size	Fear level*
g. -327 A>G	AA	96	2.3594 ± 0.1273
	AG	150	2.6133 ± 0.1105
	GG	71	2.6901 ± 0.1612
g. -125 C>T	CC	88	2.2955 ± 0.1401^b^
	CT	172	2.5814 ± 0.1026 ^ab^
	TT	57	2.8684 ± 0.1625^a^

*Values with different lowercase superscripts in the same column are significantly different (*P <* 0.05).

### Association between *STMN1* haplotype combinations and fear behavior in English Springer Spaniels

Four haplotypes were constructed from the two detected SNPs: H1 (AC), H2 (AT), H3 (GC), and H4 (GT). Consequently, there are nine combinations for the population analysis: H1H1 (ACAC), H1H2 (ACAT), H1H3 (ACGC), H1H4/H2H3 (ACGT/ATGC), H2H2 (ATAT), H2H4 (ATGT), H3H3 (GCGC), H3H4 (GCGT), and H4H4 (GTGT). The effects of the nine haplotype combinations on fear level are summarized in Table 4. The results show that fear level in dogs with the H4H4 (GTGT) haplotype combination was dramatically higher than that of dogs with any of the other haplotype combinations.

**Table 4:** LSM ± standard error for the fear level of different *STMN1* haplotype combinations in English Springer Spaniel.

Haplotype*	Sample size	Fear levels**
H1H1	32	2.1875 ± 0.2265^b^
H1H2	43	2.3721 ± 0.1941^b^
H1H3	39	2.4359 ± 0.2167^b^
H1H4/H2H3	87	2.6782 ± 0.1501^b^
H2H2	21	2.5952 ± 0.2527^b^
H2H4	24	2.6667 ± 0.2457^b^
H3H3	17	2.1765 ± 0.3235^b^
H3H4	42	2.5952 ± 0.2018^b^
H4H4	12	3.7500 ± 0.3046^a^

*H1 = AC; H2 = AT; H3 = GC; H4 = GT.

**Values with different lowercase superscripts in the same column are significantly different (*P <* 0.05).

### Promoter activity of the *STMN1* transcriptional regulatory region

**Fig 3:**
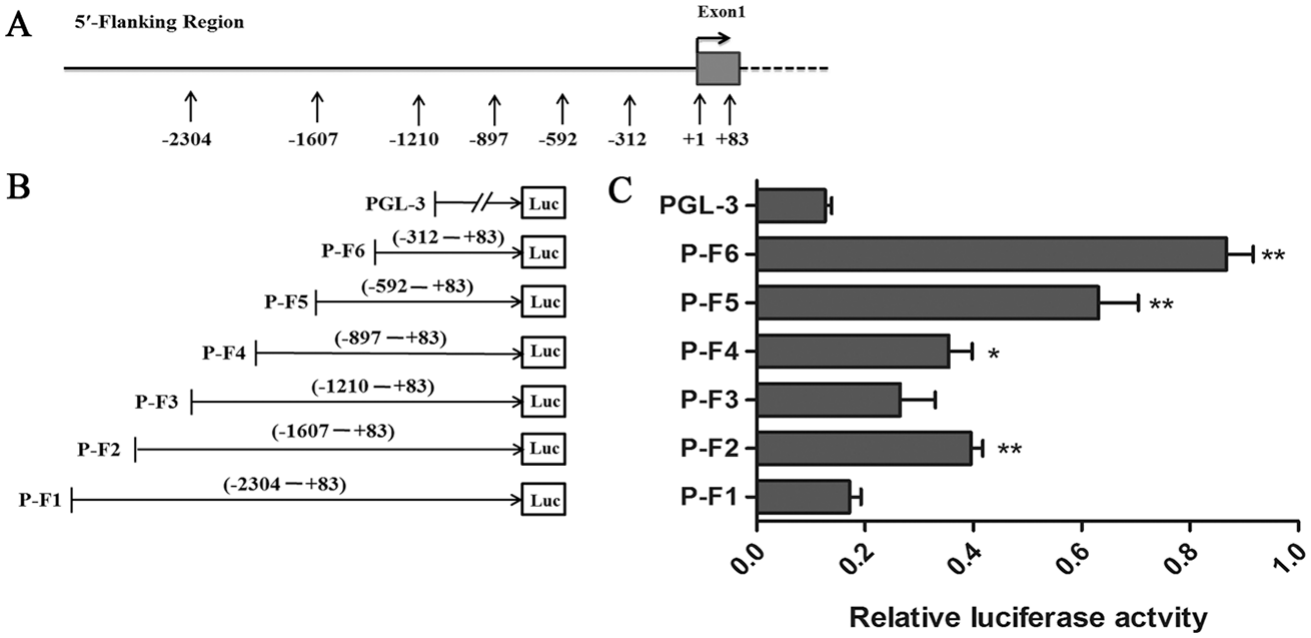
Scheme of the *STMN1* gene 5′-flanking region and identification of the core promoter region. (A) 5′-flanking region of canine *STMN1*, as identified in the National Center for Biotechnology Information (NCBI) database. Grey box represents exon 1. Other numbers represent primer positions for cloning reporter constructs. (B) Fragments P-F1, P-F2, P-F3, P-F4, P-F5, and P-F6 were amplified by PCR to produce the reporter constructs; their positions are shown in parentheses. (C) Relative luciferase activity of a series of truncated constructs in the STMN1 5′-flanking region, as measured by dual luciferase assays in 293T cells. Results are firefly luciferase activity normalized to *Renilla* luciferase activity for each sample. The pGL3-basic reporter vector was used as a control. *P < 0.05; **P < 0.01.

To determine whether the −2,304 nt to +83 nt fragment had an active promoter, we amplified this 2,387 bp fragment containing the *STMN1* transcriptional regulatory region (Fig 3A). Subsequently, we generated truncated constructs (P-F1, P-F2, P-F3, P-4, P-F5, and P-F6) by progressive deletion of nucleotides from the 5′-end, cloned these fragments into the pGL3-basic luciferase vector (Fig 3B), and transiently transfected them into 293T cells. As shown in Fig 3C, the promoter activity of all other constructs, except that of P-F1 and P-F3, was dramatically higher than that of the control pGL3 vector, and P-F6 was the highest. This result indicates that the fragment from -312 nt to +83 nt determines most of the *STMN1*promoter activity. Therefore, the P-F6 fragment of the *STMN1* proximal transcriptional regulatory region was the active core promoter. The P-F6 fragment only had g. -125 C>T, whereas the P-F5 fragment had both g. -327 A>G and g. -125 C>T. Of note, P-F5 promoter activity was lower than that of P-F6 and was significantly higher than that of all other constructs.

### Different transcriptional activity among the *STMN1* promoter haplotypes

**Fig 4:**
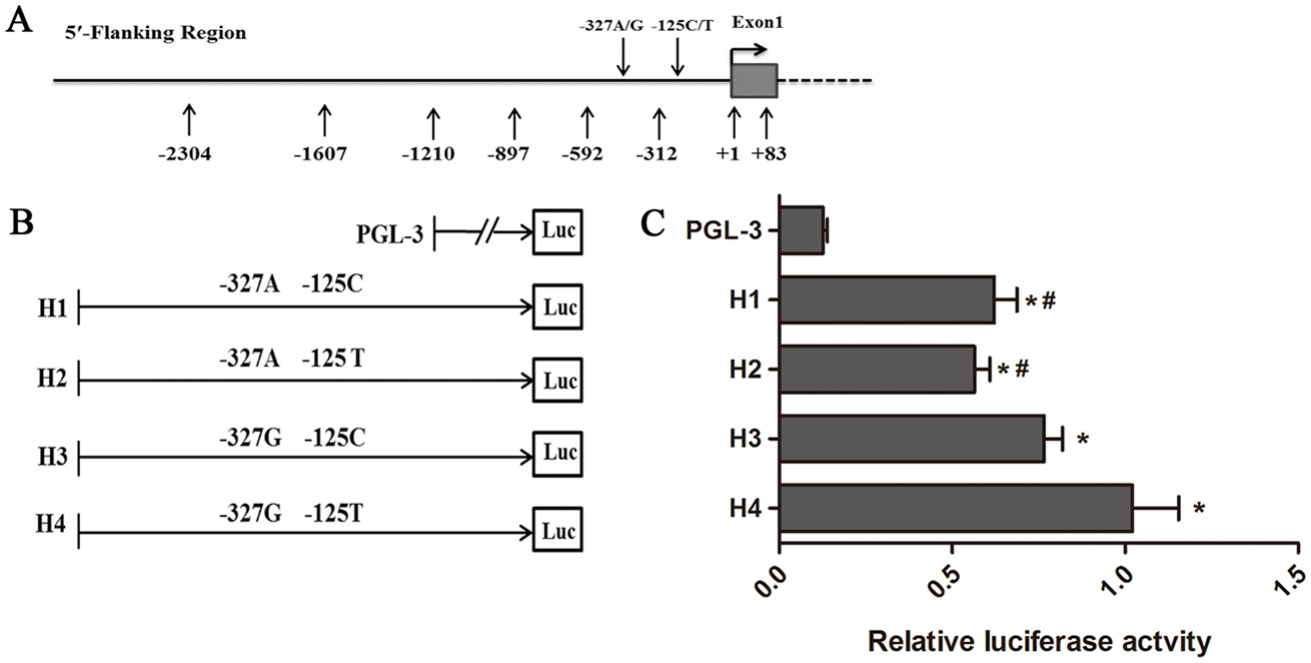
*STMN1* promoter transcriptional activity and the recombinant haplotypes. (A) 5′-flanking region of canine *STMN1*, as identified using the NCBI database. Grey box represents exon 1. g. -327 A>G and g. -125 C>T are located in the *STMN1* 5′-flanking region (−2,304 nt to +83 nt). Other numbers represent primer positions for the cloning reporter constructs. (B) The P-F5 fragment with different haplotypes (H1, H2, H3, and H4) was amplified by polymerase chain reaction to generate the reporter constructs; the various recombinant haplotypes are shown above the line. Each fragment was cloned into the pGL3 basic vector and transfected into 293T cells. (C) *STMN1* promoter transcriptional activities with various haplotypes were measured by dual luciferase assays. Results are normalized firefly luciferase activity to Renilla luciferase activity for each sample. The pGL3-basic reporter vector was used as a control. Compared with the basal activity of control, the promoter activity of all haplotype constructs was higher (*P < 0.05). Compared with the H4 activity, H1 and H2 showed significant lower activity (#*P <* 0.05).

To investigate the impact of the potential functional SNPs on *STMN1* expression, the *STMN1* promoter containing g. -327 A>G and g. -125 C>T was further analyzed on promoter activity. Four haplotype constructs (H1: AC, H2: AT, H3: GC, and H4: GT) were used to assess the effect of different promoter haplotypes on STMN1 transcriptional activity (Fig 4A and 4B). The different haplotype constructs were transiently transfected into 293T cells, withthe pGL3-basic vector as the control. As shown in Fig 4C, all constructs exhibited higher levels of luciferase expression than the control (*P* < 0.05). The H4 haplotype showed 81% and 64% higher transcriptional activity than the haplotypes H2 and H1 (*P <* 0.05), but there was no significant difference between the H4 haplotype and H3 haplotype. Given that the -592 nt and +83 nt region is the core promoter, we speculate that g. -327 A>G and g. -125 C>T may be related in transcriptional regulation of *STMN1*. Overall, our results indicate that the functional g. -327 A>G and g. -125 C>T variants in the recombined haplotypes play crucial roles in *STMN1* promoter.

### Transcription factor prediction analysis for the *STMN1* core promoter region

**Fig 5:**
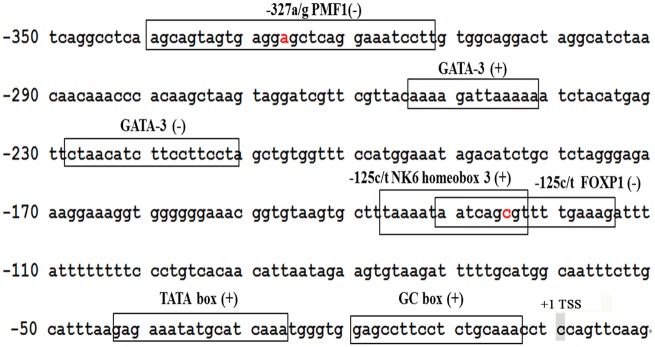
*STMN1* 5′-flanking sequence of canine from −350 nt to +10 nt. Boxed sequences represent the putative transcription factor binding sites. The presence of the g. -327 A>G mutant G allele created a *PMF1* (-) transcription factor binding motif. The presence of the g. -125 C>T mutant T allele created the *Nkx6*.*3* (+) and *FOXP1* (-) transcription factors binding motifs. Nucleotides highlighted in red represent the two polymorphic sites in English Springer Spaniels. The nucleotide sequence numbered +1 is the C of the transcription start site.

The bioinformatics and prediction analysis indicated that the *STMN1* core promoter region was located in the -477 nt to +139 nt region. The transcription factor prediction analysis showed that canine *STMN1* has a typical TATA box in its promoter region. The putative promoter contained several functional elements, including *POU6F1*, *SRY*, *c-Ets-2*, *GATA-3*, *SP2*, *MZF1*, and *E2F7* transcription factor binding sites. Both g. -327 A>G and g. -125 C>T located in the transcription factor binding site of the promoter core region. However, the presence of a mutant G allele at g. -327 A>G created the polyamine-modulated factor 1 (*PMF1*) (-) transcription factor binding motif, whereas the presence of a mutant T allele at g. -125 C>T created NK6 homeobox 3 (*Nkx6*.*3*) (+) and Forkhead Box P 1 (*FOXP1*) (-) transcription factor binding motifs (Fig 5).

**Fig 6:**
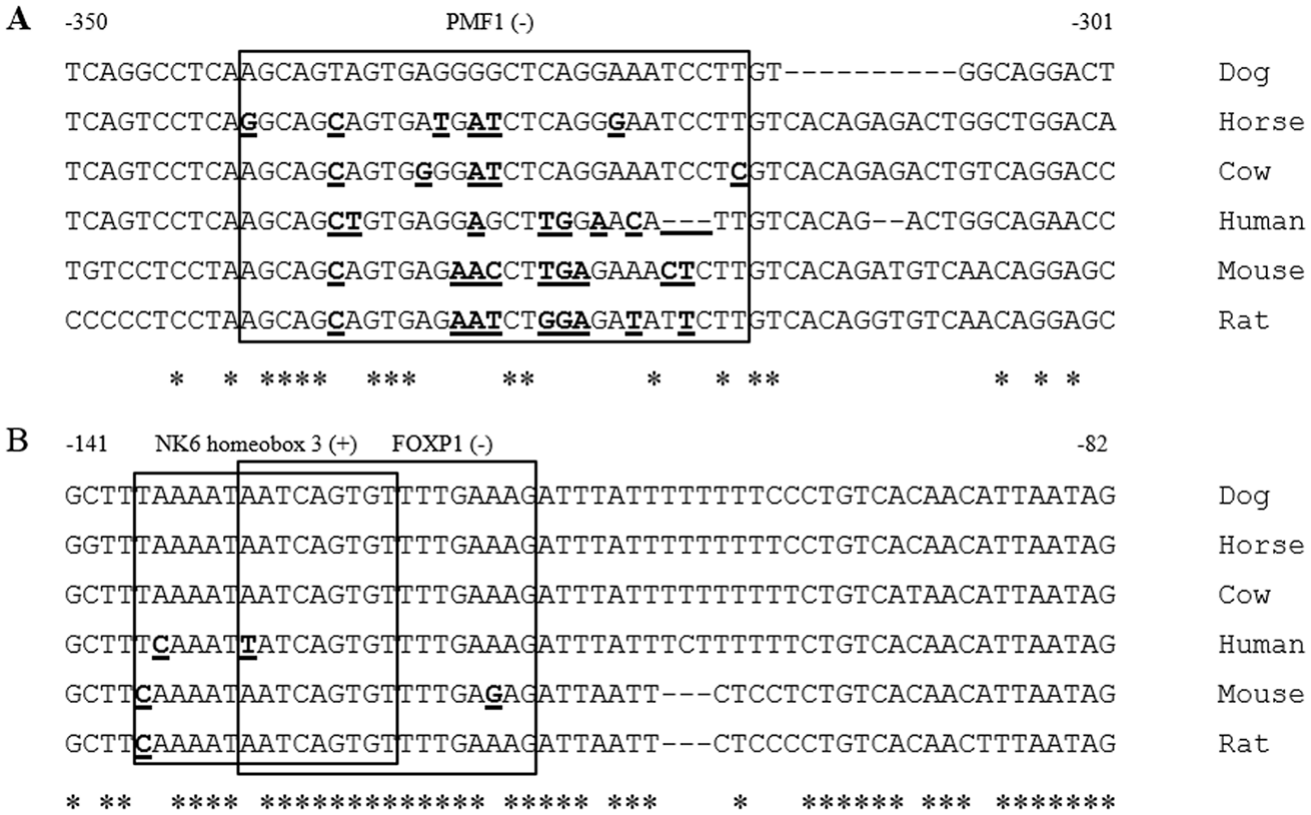
Multiple sequence alignments of the *STMN1* core promoter region among six species. (A) The black box presents *PMF1* binding site. (B) The two black boxes present *Nkx6*.*3* and *FOXP1* binding site respectively. Nucleotides are numbered relative to the dog *STMN1* gene transcription start site. Sequence consistent with dog *STMN1* is labeled by asterisk, and a gap is represented by a hyphen (-). Nucleotides shown in bold and underlined font represent differences from the dog regulatory elements.

Finally, we conducted multiple sequence alignments to determine whether the *STMN1* core promoter regions are evolutionally conserved across 6 species using publically available DNA sequences. The results revealed that the *PMF1* binding site, which is present only in dog, is poorly conserved. The *Nkx6*.*3* (+) motif is conserved in dog, horse and cow. The *FOXP1* (-) binding site is conserved in dog, horse, cow and rat. We found that human and rodent species possessed very low levels of homology with dog in the three interest binding sites (Fig 6).

## Discussion

*STMN1* can bind to tubulin, inhibit MT assembly and promote MT catastrophes, which are related to fear behavior [27, 29, 31]. *STMN1* is ubiquitously expressed in the rat brain and spinal cord, particularly within the pyriform, cingulate, and neocortex [40]. In adult rodents, *STMN1* is highly expressed in the prefrontal cortex and nucleus accumbens [41], as well as the LA and related thalamo-cortical structures [31]. Therefore, we propose that *STMN1* can affect fear behavior in dogs. To verify our hypothesis, RT-qPCR analyses were conducted to explore the distribution of the *STMN1* in 18 tissues of English Springer Spaniel. The highest *STMN1* transcript levels in our samples were found in the amygdala, which is consistent with a previous study [31]. Moreover, we detected a significant positive correlation between *STMN1* mRNA level and fear behavior, which is also in accordance with a previous report [42]. Taken together, these data suggest that *STMN1* is an important determinant of fear behavior in English Springer Spaniels.

As *STMN1* expression determined fear behavior, we hypothesized that mutations in the *STMN1* promoter region would affect *STMN1* expression differently between groups with high- and low-fear levels. The *STMN1* rs182455 SNP, which is located within or near to the putative transcriptional control region, has been previously correlated with fear responses in healthy people [27]. Moreover, the *STMN1* rs182455 C-allele was linked to cognitive-affective processing in healthy people [28]. In our study, we cloned and sequenced the transcriptional regulatory region of canine *STMN1* to determine genetic variations. Two SNPs (g. -327 A>G and g. -125 C>T) were identified in the *STMN1*promoter region, and animals with g. -125 C>T TT genotype had greater fear levels than those with the CC genotype by association analysis. The g. -125 C>T TT genotype was significantly associated with fear level, whereas the g. -327 A>G GG genotype was not. However, dogs with the H4H4 (H4 = GT) haplotype combination showed relatively higher fear levels. Therefore, the influences of the g. -327 A>G and g. -125 C>T SNPs, specifically the H4H4 haplotype combination, on fear level requires further elucidation. Overall, these results suggest that *STMN1* gene is associated with canine fear behavior, and the mutation could be used as the molecular genetic marker to select working dogs and breeding dog with low fear behavior.

We explored the transcriptional activity of the *STMN1* promoter region, and our results revealed that the P-F6 fragment had the highest promoter activity, which is in accordance with predictions of bioinformatic analysis. As the function of this fragment remains unclear, we speculate that upstream regulatory elements may interact with each other to regulate *STMN1* expression. In addition, identifying other signals that indirectly regulate *STMN1* promoter activity would be interesting. Haplotypes are more likely to affect traits than SNP [43, 44]. In this study, the g. -327 A>G and g. -125 C>T SNPs were located at the *STMN1*core promoter region, indicating their possible important function in canine *STMN1* expression. The H1 (AC), H2 (AT), H3 (GC), and H4 (GT) haplotypes were transfected into 293T cells to examine their impacts on *STMN1* promoter transcriptional activity. We found that the pGL3-H4 reporter plasmid had the highest relative luciferase activity among all plasmids. The fact that different haplotypes exhibited different promoter activities, suggests that SNPs may regulate *STMN1* expression and further affect the physiological function of *STMN1* on fear behavior.

Interestingly, the g. -327 A>G and g. -125 C>T SNPs locate in the putative canine *STMN1* promoter region. SNPs in gene coding regions can change the biological character of the encoded protein, while SNPs in non-coding regions may regulate gene expression in an allele-specific manner, and these regulatory polymorphisms stand forcrc a critical but relatively unexplored class of genetic variation. In fact, many reports found that promoter region polymorphisms regulate gene expression levels [27, 45]. Genetic variations in transcription factor binding sites may alter the binding affinity of transcription factors that control gene expression [46] and hence cause significant phenotypic diversity [47].

In our study, the existence of the mutant G allele at g. -327 A>G generated a *PMF1* (-) transcription factor binding motif, and the existence of the mutant T allele at g. -125 C>T generated the *Nkx6*.*3* (+) and *FOXP1* (-) transcription factors binding motifs. *PMF-1* doesn’t belong to Maf family, but does possess a DNA binding region and a binding site for the *NF-E2* related factor-2 (*Nrf-2*) protein [48]. *PMF-1* binds *Nrf-2* to regulate transcription of the spermidine/spermine N"-acetyltransferase gene and the *4E-BP1* gene [49, 50]. *Nkx6*.*3*, a member of the *NKX6* subfamily, contains an Engrailed-homology domain that may mediate interactions with transcriptional co-repressors [51]. Studies have revealed that it is involved in development of the central nervous system (CNS) [52, 53]. *FOXP1* and *FOXP2* belong to the *FOXP* subfamily of transcription factors [54]. They form heterodimers to control transcription [55] and are co-expressed in the brain [56, 57], suggesting that they cooperate in common pathways of cognitive and language development. Collectively, we speculate that the g. -327 A>G and g. -125 C>T mutations in the *STMN1* promoter region resulted in recruitment of different transcription factors, which subsequently altered gene expression. However, no direct evidence shows that these predicted transcription factors affect fear behavior. Further research is needed to reveal the molecular mechanisms involved.

In summary, our results suggest that the g. -327 A>G and g. -125 C>T SNPs of *STMN1* and the H4H4 (H4 = GT) haplotype combination were associated with canine fear behavior. They were identified in the putative promoter region and affected the *STMN1* transcription factor binding sites. Moreover, promoter regions with different haplotypes displayed different promoter activities, suggesting that these SNPs likely modulate *STMN1* promoter binding activity and further affect canine fear level. Overall, our data suggest that variations in *STMN1* affect the molecular signaling that regulates fear behavior. In addition, our findings demonstrate that the *STMN1* genotype is related to the regulation of fear level in canines and the two SNPs might be used as molecular markers to select working dog and breeding dog.

## Supporting Information

S1 Table The results of fear behavioral test and SNPs genotyping in 317 English Springer Spaniels. (XLSX)
